# Identification of safe channels for screws in the anterior pelvic ring fixation system

**DOI:** 10.1186/s13018-022-03191-5

**Published:** 2022-06-11

**Authors:** Lin Liu, Shicai Fan, Donggui Zeng, Hui Song, Letian Zeng, Xiangyuan Wen, Dadi Jin

**Affiliations:** 1 Orthopedic Trauma, University of Chinese Academy of Sciences Shenzhen Hospital, Shenzhen, Guangdong People’s Republic of China; 2 The Third Affiliated Hospital, Southern Medical University, Guangzhou, Guangdong People’s Republic of China

**Keywords:** Anterior pelvic ring fixation system, Pelvic fracture, Safe channel, Minimally invasive

## Abstract

**Background:**

Minimally invasive surgery for pelvic fracture using anterior ring internal fixator system is increasing gradually, and the way to insert the fixation screws in the fixation system is the key technical points of the method. However, there have been few studies on insertion of fixation screws for the anterior pelvic ring internal fixator system.

**Objective:**

To identify safe channels for fixation screws in the anterior pelvic fixator system and provide the anatomical basis for insertion of fixation screws in clinical operation.

**Methods:**

Screw insertion was simulated into a total of 40 pelvic finite element models as well as 16 fresh pelvic specimens, and the channel parameters were measured.

**Results:**

Finite elements (male, female) include: screws in ilium: length 114.4 ± 4.1 and 107.6 ± 8.3 mm, respectively; diameter 11.7 ± 0.5 and 10.0 ± 0.6 mm, distance between screw and anterior inferior iliac spine: 5.5 ± 1.0 and 5.6 ± 1.0 mm, angle of coronal plane 55.8° ± 2.4° and 50.6° ± 3.1°, angle of sagittal plane 26.6° ± 1.0° and 24.5° ± 1.9° and angle of horizontal plane 64.9 ± 3.7 and 58.1 ± 3.1; screws in pubis: length 47.0 ± 2.0 and 39.8 ± 3.9 mm, diameter 7.1 ± 0.4 and 6.1 ± 0.4 mm. Specimens (male, female) include: distance between screw and anterior inferior iliac spine: 5.5 ± 0.5 and 5.6 ± 0.7 mm, angle of coronal plane 55.9° ± 1.3° and 50.7° ± 1.5°, angle of sagittal plane 26.7° ± 0.5° and 24.1° ± 0.9° and angle of horizontal plane 64.8° ± 0.6° and 58.8° ± 0.8°. In the comparison between female and male in each group, differences in distances between screws and anterior inferior iliac spine and median line of symphysis pubis (*P* > 0.05) were not statistically significant; differences in the remaining parameters were statistically significant (*P* < 0.05).

**Conclusions:**

If surgeons paid attention to sex differences, select screws of appropriate diameter and length and hold the insertion position and direction, screws in the anterior pelvic ring fixation system could be safely inserted.

## Background

With the development of minimally invasive technologies for the treatment of pelvic fracture, good outcomes for fractures of the anterior pelvic ring treated with a subcutaneous fixator [1, 2] or INFIX technology [3, 4] have been reported. However, the best way to insert the fixation screws in the fixation system, one of the key technical points of the method, has rarely been reported. Failure of fixation of the pelvic fracture can result from improper placement of fixation screws. Screw penetration of the bone cortex may damage the lumbosacral plexus nerves, internal iliac vessels, genitourinary system and gastrointestinal system, and due to significant blood loss and disruption of the lumbosacral plexus, genitourinary system and gastrointestinal system, severe consequences of massive hemorrhage may lead to death [5]. Injury of lumbosacral plexus nerves and genitourinary system may lead to sexual dysfunctions with important influence on quality of life, especially in young patients [6].

With the development of orthopedic navigation technology, some scholars have applied navigation technology to insert fixation screws in pelvic fractures [7]. Ciolli et al. inserted percutaneous screws of the anterior ring internal fixator system by navigation system based on 3D fluoroscopic images, and the conclusion is that the use of the O-arm deals with great precision in the positioning of the screw and reduced surgical times with excellent clinical results in patients [6]. However, navigation technology requires special intraoperative imaging system, and surgery requires longer learning curves. Is there a suitable and simple method to insert fixed screws safely and quickly? In this study, the parameters of safe screw channels were investigated using the finite element method and the traditional anatomic method to provide the anatomical basis for best clinical practice.

## Materials and methods

### Materials

We used original image data of normal pelvises scanned with a Toshiba/Aquilion 64-row 128-slice spiral CT (Toshiba, Japan) in the Third Affiliated Hospital of Southern Medical University or a Siemens Somatom Definition Flash dual-source 64-row 128-slice CT (Siemens AG, Erlangen, Germany) in the University of the Chinese Academy of Sciences Shenzhen Hospital between January 2017 and January 2020, comprising 20 males and 20 females, aged 20–65 years with a mean age of 42 years.

In addition, the Department of Anatomy of Southern Medical University provided 16 fresh and cold adult pelvic specimens (10 male specimens and six female specimens, aged 25–66 years with a mean age of 43.5 years), which were free of fractures, tumors, malformations and obvious osteoporosis as ascertained by visual observation and CT examination. Each of the specimens was a complete pelvis including the L5 vertebral column.

### Finite element research method

**Fig. 1:**
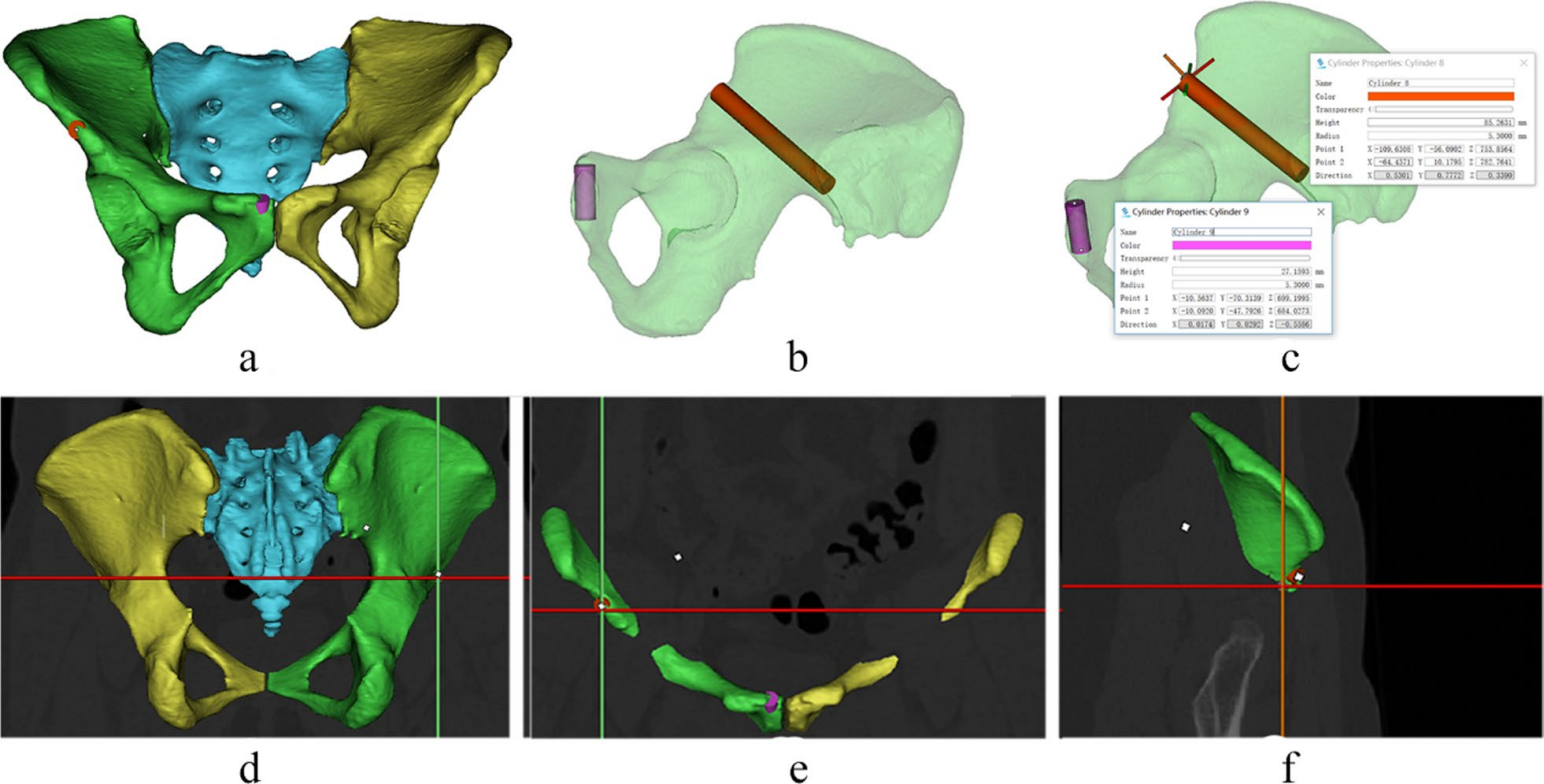
Model-simulated screw insertion and measurement; **a** insertion position, **b** insertion channel, **c** parameter measurement, **d** observation in horizontal plane, **e** observation in coronal plane, **f** observation in sagittal plane

CT image data were imported into Mimics 21.0 (Materialise, Leuven, Belgium) to create finite element pelvic model images [8, 9], and a cylinder was created to simulate the insertion of a fixation screw from the superior margin of the anterior inferior iliac spine to the posterior inferior iliac spine and another screw insertion in the para-pubic region perpendicular to the surface of the pubis. The axis of each cylinder was defined as the best approach, and the radius of each cylinder was adjusted, from small to large. Observations were taken from the coronal plane, sagittal plane and horizontal plane, and in these three 2D planes, if the cylinder protruded either side of the bone cortex, the face where this point was located was defined as the narrowest point of the fixation screw channel [10, 11]. If the screws did not break through the cortical bone or out of the joint, parameters such as the diameter and length of the cylinder, distance from the anterior inferior iliac spine or the symphysis pubis and angles between the screws and coronal, sagittal and horizontal planes of the human body were measured (Fig. 1).

### Specimen study methods

**Fig. 2:**
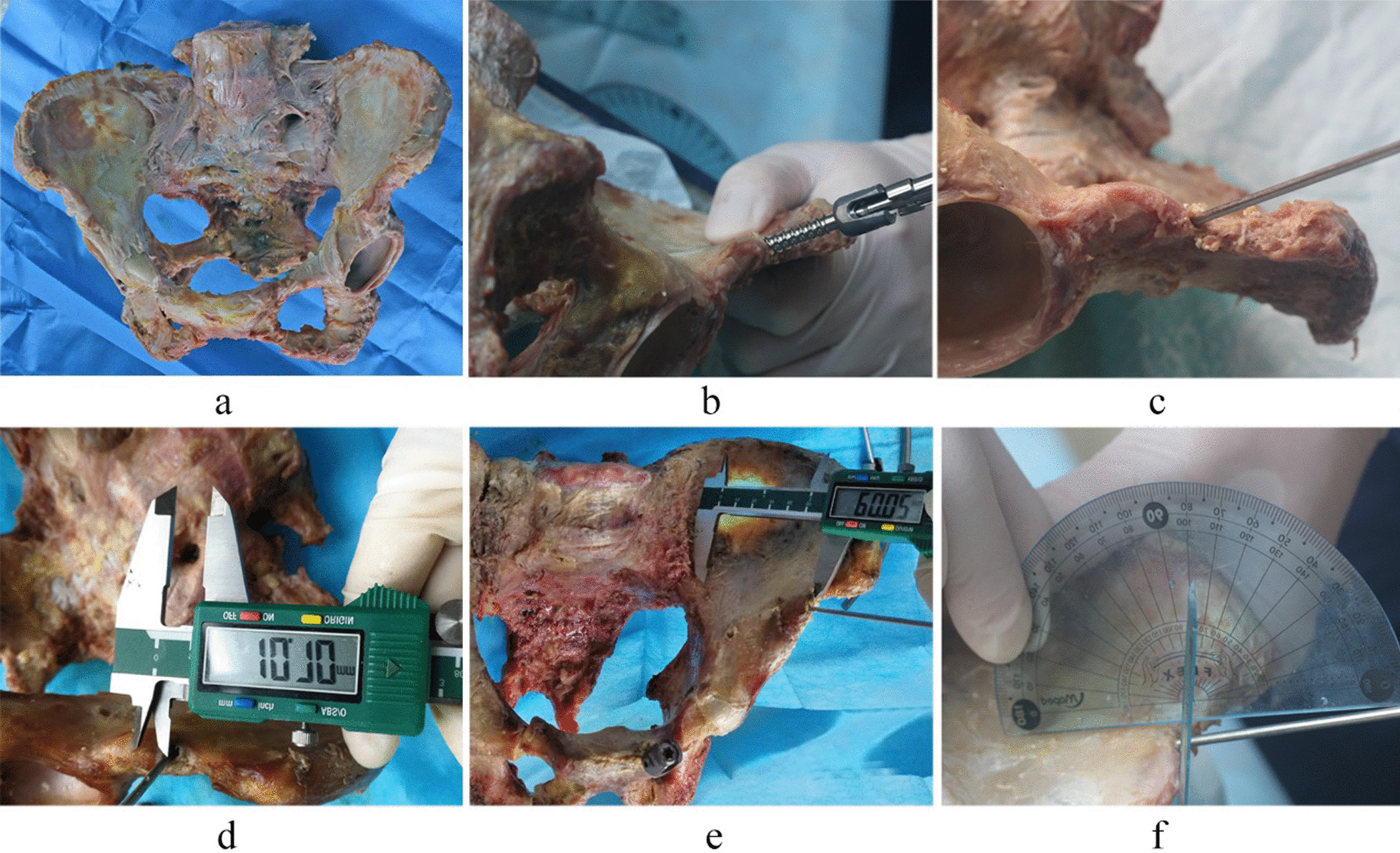
Measurement of screw parameters in specimens; **a** pelvis specimen, **b** screw insertion, **c** Kirschner wire insertion, **d** distance measurement, **e** length measurement, **f** angle measurement

Several leads with a diameter of 2.5 mm were inserted into the anterior inferior iliac spine approximately 5 mm from the superior margin of the anterior inferior iliac spine [12] to determine the optimal insertion point and placement position of the screws, and a screw channel between the internal and external plates of the ilium whose depth should be measured was created. Then, a titanium alloy universal fixation screw of the anterior ring (Xiamen Double Medical Co., Ltd., Xiamen, China) of 7.5 mm in diameter and 60–90 mm in length was inserted with the screw tail exposed 10–15 mm outside the bone. In the 1 cm area between the pubis and the symphysis pubis, a universal fixation screw of the anterior ring with a diameter of 45 mm and a length of 35–45 mm was inserted and according to our observation, the position of the screw was correct with no protrusion from the bone cortex. The screw was then removed, and a Kirschner wire with diameter of 2.5 mm was inserted to measure the distance between the screw and the anterior inferior iliac spine and the symphysis pubis, as well as the angle between the screws in the ilium and the anatomic planes (Fig. 2).

### Statistical methods

SPSS 21.0 software (IBM SPSS Statistics for Windows, Armonk, NY, USA) was used for statistical analysis of the data. All measurement data are expressed as ${\overline{\text{x}}} \pm {\text{s}}$ and compared by paired *t* test. A difference was considered statistically significant if *P* < 0.05.

## Results

### Experimental results obtained by the finite element method

**Fig. 3:**
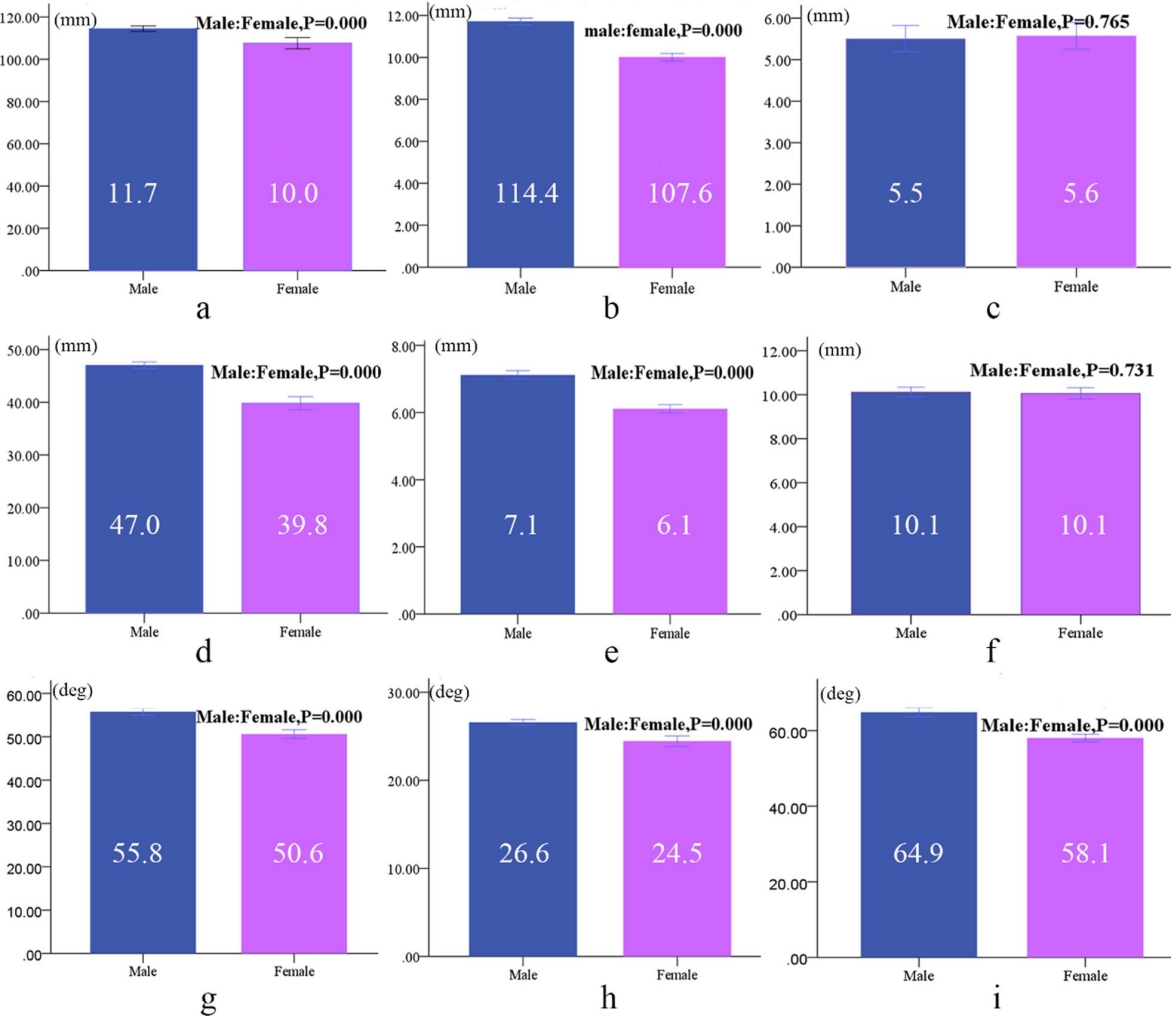
Parameters of screws in finite element model (*n* = 40); **a** length of screw in ilium, **b** diameter of screw in ilium, **c** distance between screw and anterior inferior iliac spine, **d** length of screw in pubis, **e** diameter of screw in pubis and **f** distance between screw and symphysis pubis, **h** included angle between screw and coronal plane, **i** included angle between screw and sagittal plane, **g** included angle between screw and horizontal plane

The maximum length and diameter of the screws, the distance between the screw in the ilium and the anterior inferior iliac spine and the distance between the screw in the pubis and the symphysis pubis are shown in Table 1. Left-and-right comparison showed that differences between the data of the same sex (*P* > 0.05) were not statistically significant. Ignoring differences between data from left- and right-side screws in the ilium of males and screws in the ilium of females: with maximum length of 114.4 ± 4.1 mm and 107.6 ± 8.3 mm, respectively, and maximum diameter of 11.7 ± 0.5 and 10.0 ± 0.6 mm, the distance from the symphysis pubis was 5.5 ± 1.0 and 5.6 ± 1.0 mm. In comparison between males and females, the differences in the length and diameter of the screws were statistically significant (*P* < 0.05) (Fig. 3a, b), but the differences in distance between the screws and the symphysis pubis were not statistically significant (*P* > 0.05) (Fig. 3c). Regarding screws in the pubis of females and males: with maximum lengths of 47.0 ± 2.0 and 39.8 ± 3.8 mm, maximum diameters of 7.1 ± 0.4 and 6.1 ± 0.4 mm, the distances between the screws and the symphysis pubis were 10.1 ± 0.7 and 10.1 ± 0.8 mm, respectively. When compared between males and females, the differences in the length and diameter of the screws were statistically significant (*P* < 0.05) (Fig. 3d, e) and the differences in distance between the screws and the symphysis pubis were not statistically significant (*P* > 0.05) (Fig. 3f).

**Table 1:** The length, diameter and position parameters of screws (mm, *n* = 20)

Parameters	Male		Female	
	Left	Right	Left	Right
Length of screw in ilium	114.6 ± 4.2	114.3 ± 4.0	107.7 ± 9.7	107.5 ± 7.0
Diameter of screw in ilium	11.7 ± 0.5	11.7 ± 0.5	10.0 ± 0.6	10.0 ± 0.6
Distance between screw and anterior inferior iliac spine	5.5 ± 1.0	5.5 ± 1.0	5.6 ± 0.9	5.6 ± 1.2
Length of screw in pubis	46.9 ± 1.1	47.2 ± 2.6	39.9 ± 3.2	39.8 ± 4.5
Diameter of screw in pubis	7.1 ± 0.3	7.1 ± 0.5	6.1 ± 0.3	6.1 ± 0.4
Distance between screw and symphysis pubis	10.1 ± 0.7	10.1 ± 0.7	10.1 ± 0.7	10.1 ± 0.9

Left-and-right comparison of same-sex data indicated that the differences were not statistically significant

The angle between the screws in the ilium and the coronal, sagittal and horizontal planes of the human body is shown in Table 2. The left-and-right comparison showed that differences in same-sex data were not statistically significant (*P* > 0.05). Ignoring the differences between data of left and right sides, the included angles between screws in the ilium and the coronal plane were 55.8° ± 2.4° and 50.6° ± 3.1°, and those in the sagittal plane were 26.6° ± 1.0° and 24.5° ± 1.9°, while those in the horizontal plane were 64.9° ± 3.7° and 58.1° ± 3.1°. The differences in the included angles between males and females were statistically significant (*P* < 0.05) (Fig. 3g–i).

**Table 2:** Orientation parameters of the screw in the ilium (degrees, n = 20)

Parameters	Male		Female	
	Left	Right	Left	Right
Angle of coronal plane	55.8 ± 2.4	55.7 ± 2.6	50.9 ± 3.6	50.3 ± 2.4
Angle of sagittal plane	26.6 ± 1.2	26.6 ± 0.9	24.6 ± 1.9	24.3 ± 1.9
Angle of horizontal plane	64.6 ± 1.2	64.9 ± 4.4	58.1 ± 3.3	58.0 ± 3.0

The left-and-right comparison showed that none of the differences in any parameters were statistically significant (*P* > 0.05)

### Results of the study of pelvic specimens

**Fig. 4:**
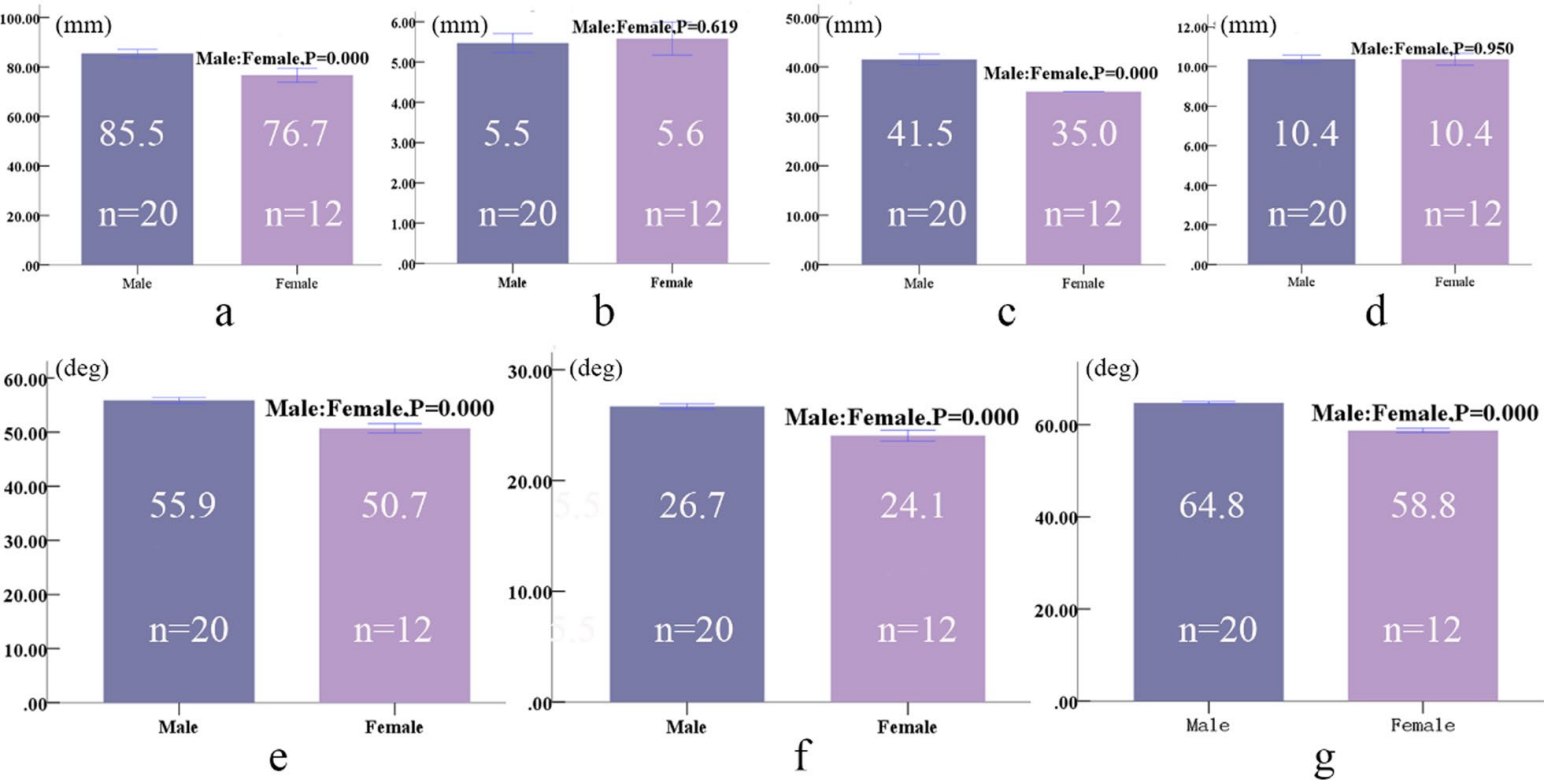
Parameters of screws in specimens (male 20, female 12); **a** length of screw in ilium, **b** distance between screw and anterior inferior iliac spine, **c** length of screw in pubis, **d** distance between screw and symphysis pubis, **e** included angle between screw in ilium and coronal plane, **f** included angle between screw and sagittal plane, **g** included angle between screw and horizontal plane

The diameters of the screws in the ilium and pubis were 7.5 mm and 4.5 mm, respectively, and the screws in the pubis were inserted perpendicular to the upper surface of the pubis. The position and length of screws are shown in Table 3, and the angles between screws in the ilium and the anatomic planes are shown in Table 4. The left-and-right comparison showed that differences of same-sex data were not statistically significant (*P* > 0.05). Ignoring left-and-right differences of the data, the lengths of screws in the ilium in males and females were 85.5 ± 3.6 and 76.7 ± 4.9 mm and the lengths of screws in the pubis were 41.5 ± 2.4 and 35.0 ± 0.0 mm. The difference between females and males was statistically significant (*P* < 0.01) (Fig. 4a, c). In males and females, the distances between the screws and the anterior inferior iliac spine were 5.5 ± 0.5 and 5.6 ± 0.7 mm and the distances between the screws and the symphysis pubis were 10.4 ± 0.4 and 10.4 ± 0.5 mm. The differences between females and males were not statistically significant (*P* > 0.05) (Fig. 4b, d). In females and males, the included angles between screws in the ilium and the coronal plane were 55.9 ± 1.3 and 50.7° ± 1.5°, the included angles between screws in the ilium and in the sagittal plane were 26.7° ± 0.5° and 24.1° ± 0.9°, and the included angles between screws in the ilium and in the horizontal plane were 64.8° ± 0.6° and 58.8° ± 0.8°, respectively. The differences between females and males were statistically significant (*P* < 0.01) (Fig. 4e-g).

**Table 3:** The position parameters of screws in pelvic specimens (mm, males 10, females 6)

Parameters	Male		Female	
	Left	Right	Left	Right
Distance between screw and anterior inferior iliac spine	5.5 ± 0.5	5.4 ± 0.6	5.6 ± 0.7	5.6 ± 0.8
Distance between screw and symphysis pubis	10.4 ± 0.4	10.4 ± 0.5	10.3 ± 0.5	10.5 ± 0.5
Length of screw in ilium	85.5 ± 3.7	85.5 ± 3.7	76.7 ± 5.2	76.7 ± 5.2
Length of screw in pubis	41.5 ± 2.4	41.5 ± 2.4	35.0 ± 0.0	35.0 ± 0.0

Left-and-right comparison showed differences in same-sex data were not statistically significant (*P* > 0.05)

**Table 4:** The orientation parameters of screws in the ilium (degrees, 10 males, 6 females)

Parameters	Male		Female	
	Left	Right	Left	Right
Angle of coronal plane	55.9 ± 1.3	55.9 ± 1.4	50.8 ± 1.6	50.7 ± 1.5
Angle of sagittal plane	26.8 ± 0.5	26.6 ± 0.5	24.1 ± 0.9	24.0 ± 0.9
Angle of horizontal plane	64.8 ± 0.6	64.7 ± 0.5	58.7 ± 0.8	58.8 ± 0.8

Left-and-right comparison showed differences in same-sex data were not statistically significant (*P* > 0.05)

### Validation of the finite element model

**Fig. 5:**
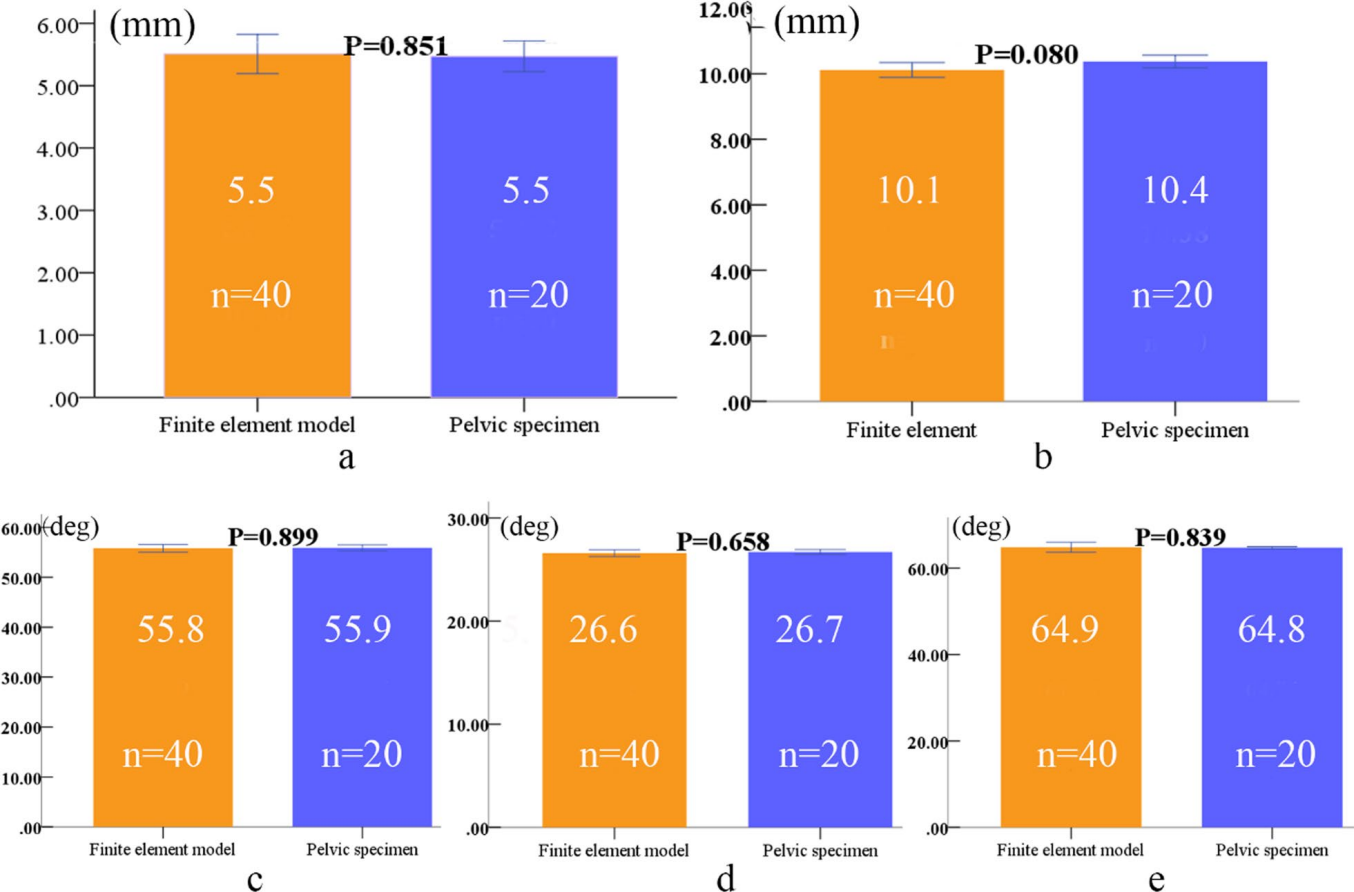
Comparison of parameters between finite element models analysis and pelvic specimens of males (finite element model 40, specimens 20); **a** distance between the screw and the anterior inferior iliac spine, **b** distance between screw and symphysis pubis, **c** included angle between screw and coronal plane, **d** included angle between screw and sagittal plane, e included angle between screw and horizontal plane

**Fig. 6:**
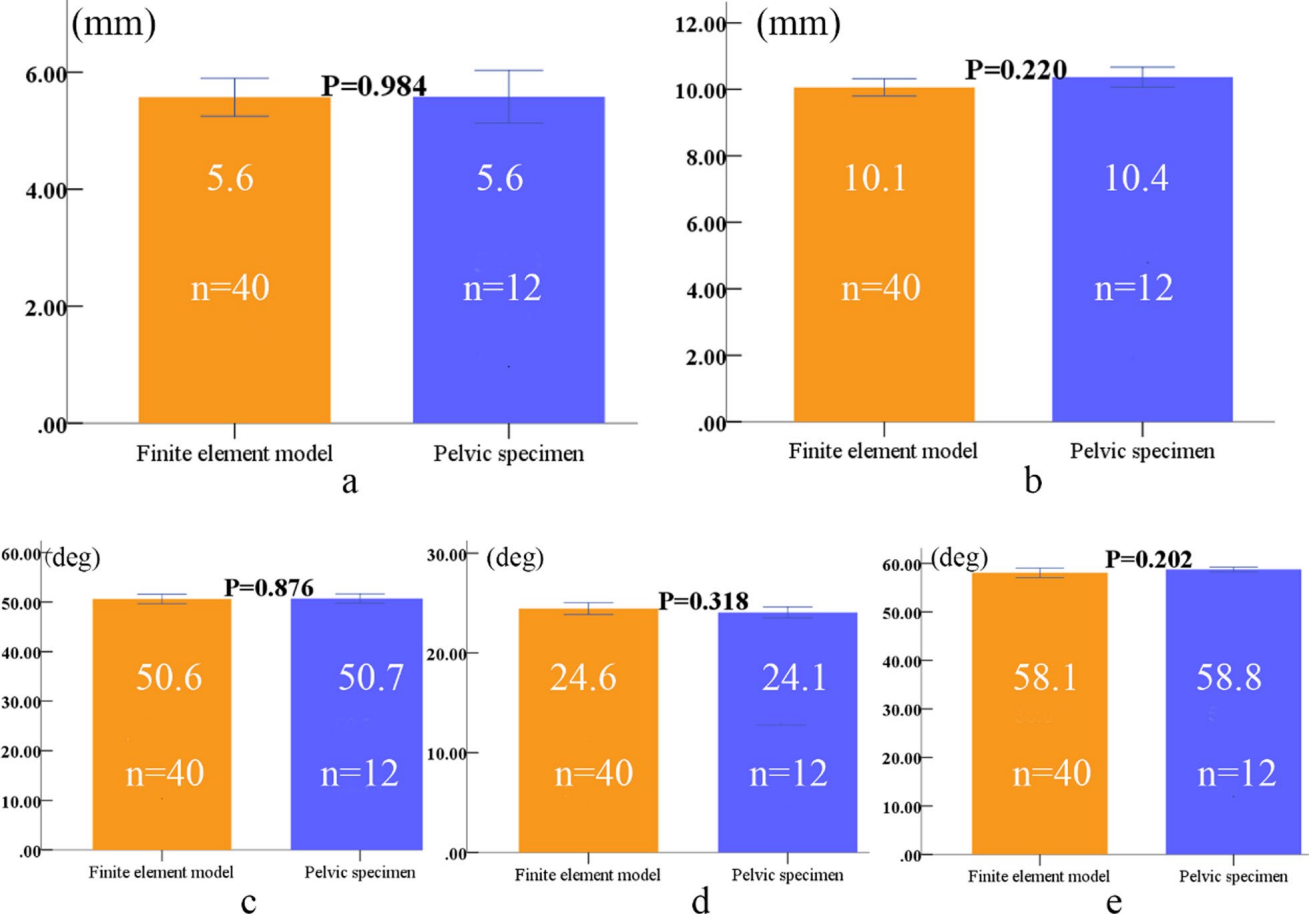
Comparison of parameters between finite element models analysis and pelvic specimens of females (finite element model 40, specimens 12); **a** distance between the screw and the anterior inferior iliac spine, **b** distance between screw and symphysis pubis, **c** included angle between screw and coronal plane, **d** included angle between screw and sagittal plane, **e** included angle between screw and horizontal plane

Differences in the distance between the screw in the ilium, those in the anterior inferior iliac spine and those in the symphysis pubis, and the included angles between the screws in the ilium and the anatomic planes between the finite element model group and the pelvic specimen group of the same sex were not statistically significant (*P* > 0.05) (Figs. 5, 6). These results show that the finite element model developed in this study was validated in terms of the channel parameters of the screw.

## Discussion

In previous studies, research on the optimal channels for pelvic screws was mainly based on traditional autopsy measurements, because they were based on actual anatomy and cannot be surpassed by other testing methods [13]. However, this approach is restricted by many factors such as source, repeatability and man-made measurement errors [14, 15]. In recent years, Mimics software has become commonly used in digital anatomical research, which transforms bones and soft tissues into virtual 3D models using CT or MRI scanning data [16]. Based on these 3D models, anatomical measurements, simulated operations and prosthesis design can then be carried out. In comparison with traditional anatomy and imaging studies, it has the advantages of low cost, high repeatability, comprehensive information acquisition and good reliability, high measurement precision and more accurate experimental results and avoids the disadvantages of the traditional autopsy method, such as limited resources, difficult operation and inaccurate results [17]. In this study, a 3D finite element model of the pelvis was established using Mimics software to simulate the internal fixation system with screws inserted. The maximum length, diameter and angle between the screw and the anatomic axis were measured. At the same time, internal screws were inserted into a few pelvic specimens and the parameters of each channel were measured to verify the accuracy of data obtained by the finite element method. The differences in the distance between the screw and the anterior inferior iliac spine and the symphysis pubis and the included angles between the screws in the ilium and the anatomic planes between the finite element models and pelvic specimens of the same sex were not statistically significant, indicating that the safe channel parameters obtained by the finite element model were accurate.

The main complications of repairing fractures of the anterior pelvic ring with a fixation system include injury to the lateral femoral cutaneous nerve and femoral nerve, with an incidence rate ranging from 4% as reported by Cole et al. [18] to 32% as reported by Kuttner et al. [19]. The reported incidence of complications in the application of the anterior pelvic ring fixation system varies greatly [20], and consequently, there have been some studies of the safety of this system. Validya et al. provided clear guidance on how to safely insert the fixation system [21]. Merriman et al. [22] considered that the average distance between the connective bar and the vascular bundle should be greater than 2.2 cm to prevent compression of the vascular nerve. Apivatthakakul et al. [23] suggested that a universal screw should be used for fixation with some space on the screw, and that the surface of the ilium and both ends of the connective bar should be as short as possible to minimize injury to the lateral femoral cutaneous nerve.

At present, studies on the safety of the internal fixation system have mainly focused on the distance of the fixation bar and the fixation screw from the bone surface and its distance from the main anatomical structures, but there have been few anatomical studies on fixation screw insertion. Safe and effective insertion of the fixation screws is the first step of a successful operation. In this study, the safe channels for inserted screws were studied using the finite element method and the traditional anatomical method to provide an anatomical basis for rapid and safe insertion of fixation screws in the clinic.

In the study, the differences in the maximum length and diameter of screws in the ilium and pubis and the angles between the ilium screw and the anatomic planes in males and females were statistically significant, while the differences in the distances between the screw and the anterior inferior iliac spine and the symphysis pubis in males and females were not statistically significant. It can be seen that due to the difference between males and females, the shape of the pelvis, the safe length, diameter and included angles between the anatomical faces and the screws differed between males and females. In the common anterior fixation system used in the clinic, fixation screws used in the ilium had a diameter of 7.5 mm and a length of 60–100 mm, while the fixation screws used in the pubis had a diameter of 4.5 mm and a length of 35–45 mm. In this study, the maximum diameter and the maximum length of the screws in the ilium in males and females were larger than 10 mm and 100 mm, respectively, the maximum diameter and the maximum length of the screws in the pubis in males and females were larger than 6 mm and 35 mm, and the length and diameter of screws inserted into males were greater than that of those inserted into females. Therefore, in clinical practice, screws in the ilium can be 7.5 mm in diameter for both men and women, screws with a length of 70–100 mm can be used for men and screws with a length of 60–90 mm can be used for women. Screws in the pubis can have a diameter of 4.5 mm for both men and women and screws with a length of 35–45 mm can be used for men, while screws 35 mm in length can be used for women. Safe insertion of the screws depends on correct position and orientation. According to our research results, there were no differences between males and females with regard to screw insertion points, which were approximately 5 mm above the anterior inferior iliac spine, and approximately 10 mm outside the midline of the symphysis pubis. The screws in the pubis needed to be inserted from top to bottom and perpendicular to the upper surface of the pubis. However, due to the irregular anatomical shape of the ilium, it was difficult to insert the screws safely, and also due to the difference in the angles between the screws and the anatomical surfaces between men and women, attention should be paid to gender differences during the operation. According to the study results, screws in the ilium could be safely inserted when the angles between the screw and the coronal, sagittal and horizontal planes are approximately 56°, 26° and 65°, respectively, in males and 51°, 25° and 59° in females. The above steps can be used to guide the rapid and safe insertion of fixation screws in the clinic, avoid the risks of a screw protruding from the cortex or the joint and damaging the pelvic organs, reduce the number of X-ray fluoroscopies and shorten the operation time.

## Deficiencies of the study

In the study, ideal conditions for screw insertion were simulated and the thickness of the iliac and pubic cortex may differ from the real situation during simulated screw insertion, with the result that the length and maximum diameter of the simulated screws may differ from screws actually inserted. In clinical surgery, we can appropriate screws by evaluating the length and diameter of screws according to preoperative image data, so that we can observe whether such defect exists and make corresponding countermeasures.

The actual insertion point and orientation of the screws may differ from those of the normal pelvis due to displacement of a pelvic fracture and changes of body position. In clinical surgery, we can confirm the insertion point and orientation of the screws.

## Future study

Our further studies will focus on clinical application of pelvic fracture treatment using an internal fixator system. Their clinical effects will be investigated.

## Conclusions

The screws in the ilium of the anterior pelvic ring fixation system (male, female) had a maximum length of 114.44 ± 4.07 and 107.59 ± 8.34 mm, maximum diameter of 11.71 ± 0.52 and 10.01 ± 0.55 mm, distance between screw and anterior inferior iliac spine of 5.51 ± 0.98 and 5.57 ± 1.01 mm, angle between the screw and the coronal plane of 55.79° ± 2.44° and 50.62° ± 3.05° and angle between the screw and sagittal plane of 26.58° ± 1.0° and 24.45° ± 1.87°, angle between the screw and horizontal plane of 64.88° ± 3.67° and 58.05° ± 3.10°; screws in pubis (male, female): maximum length of 47.02 ± 1.96 mm and 39.81 ± 3.84 mm, maximum diameter of 7.12 ± 0.38 and 6.12 ± 0.37 mm and distance between screw and symphysis pubis of 10.11 ± 0.71 and 10.06 ± 0.82 mm. According to the above data, in order to safely insert screws of the anterior pelvic ring fixation superior margin of the anterior inferior iliac spine and the symphysis pubis, attention should be paid to gender differences, selection of screws of the appropriate diameter and length and holding the position and orientation.

## Data Availability

The datasets used and analyzed in the study are available from the corresponding author on request to the corresponding author.

## Abbreviations

INFIX: Internal fixator
CT: Computed tomography
MRI: Magnetic resonance imaging
3D: Three dimensions
